# Facile Synthesis of Phosphorus and Cobalt Co-Doped Graphitic Carbon Nitride for Fire and Smoke Suppressions of Polylactide Composite

**DOI:** 10.3390/polym12051106

**Published:** 2020-05-12

**Authors:** Xianwu Cao, Xiaoning Chi, Xueqin Deng, Qijun Sun, Xianjing Gong, Bin Yu, Anthony Chun Yin Yuen, Wei Wu, Robert Kwow Yiu Li

**Affiliations:** 1 National Engineering Research Center of Novel Equipment for Polymer Processing, Key Laboratory of Polymer Processing Engineering of Ministry of Education, Guangdong Provincial Key Laboratory of Technique and Equipment for Macromolecular Advanced Manufacturing, South China University of Technology, Guangzhou 510640, China; ppeme@scut.edu.cn (X.C.); cxnnxc0402@163.com (X.C.); xueqindd@163.com (X.D.); 2 Department of Materials Science and Engineering, City University of Hong Kong, Tat Chee Avenue, Kowloon, Hong Kong SAR 999077, China; qjsun2-c@my.cityu.edu.hk (Q.S.); jingxgong2-c@my.cityu.edu.hk (X.G.); aprkyl@city.edu.hk (R.K.Y.L.); 3 Centre for Future Materials, University of Southern Queensland, Toowoomba 4350, Australia; 4 School of Mechanical and Manufacturing Engineering, University of New South Wales, Sydney 2052, Australia; c.y.yuen@unsw.edu.au

**Keywords:** polylactide, graphitic carbon nitride, flame retardancy, smoke suppression

## Abstract

Due to the unique two-dimensional structure and features of graphitic carbon nitride (g-C_3_N_4_), such as high thermal stability and superior catalytic property, it is considered to be a promising flame retardant nano-additive for polymers. Here, we reported a facile strategy to prepare cobalt/phosphorus co-doped graphitic carbon nitride (Co/P-C_3_N_4_) by a simple and scalable thermal decomposition method. The structure of Co/P-C_3_N_4_ was confirmed by scanning electron microscopy (SEM), X-ray diffraction (XRD) and X-ray photoelectron spectroscopy (XPS). The carbon atoms in g-C_3_N_4_ were most likely substituted by phosphorous atoms. The thermal stability of polylactide (PLA) composites was increased continuously with increasing the content of Co/P-C_3_N_4_. In contrast to the g-C_3_N_4_, the Polylactide (PLA) composites containing Co/P-C_3_N_4_ exhibited better flame retardant efficiency and smoke suppression. With the addition of 10 wt % Co/P-C_3_N_4_, the peak heat release rate (PHRR), carbon dioxide (CO_2_) production (PCO2P) and carbon oxide (CO) production (PCOP) values of PLA composites decreased by 22.4%, 16.2%, and 38.5%, respectively, compared to those of pure PLA, although the tensile strength of PLA composites had a slightly decrease. The char residues of Co/P-C_3_N_4_ composites had a more compact and continuous structure with few cracks. These improvements are ascribed to the physical barrier effect, as well as catalytic effects of Co/P-C_3_N_4_, which inhibit the rapid release of combustible gaseous products and suppression of toxic gases, i.e., CO.

## 1. Introduction

Polylactide (PLA) is a widespread biobased and biodegradable polyester. It has been widely utilized in numerous fields, such as agriculture, packaging, automobile components and electronics [1,2,3,4,5], due to its superior physical and mechanical properties. However, some disadvantages, like poor thermal stability, weak mechanical strength and high flammability of PLA restrict its further applications. Recently, two-dimensional (2D) nanomaterials such as graphene and its derivatives [6], montmorillonite (MMT) [7], and layered double hydroxide (LDH) [8,9], have been explored to improve the thermal stability, fire performance, and mechanical properties of PLA matrix. 

Graphitic carbon nitride (g-C_3_N_4_) is a 2D nanomaterial that has received considerable attention, due to its thermal stability, chemical resistance and unique optical properties [10]. It can be large scale prepared by rapid polymerization of cheap precursors, like cyanamide [11], melamine [12], and dicyandiamide [13]. Nowadays, many researchers have attempted to utilize g-C_3_N_4_ or its modifications as flame-retardant additives to enhance the flame retardancy of polymers [14]. Shi et al. demonstrated that the g-C_3_N_4_/MWCNT hybrid layer on the surface of PS spheres could act as “tortuous path” to reduce the permeation of heat [15]. The g-C_3_N_4_ nanosheets, in combination with reduced graphene oxide, served as excellent barriers in the PS matrix and reduced pyrolysis gaseous products during combustion [16]. Moreover, it has been demonstrated that the incorporation of various traditional flame retardants, together with g-C_3_N_4_, could endow polymers with better flame retardancy [17]. However, the poor thermal-oxidative stability and limited catalytic charring capacity of g-C_3_N_4_ limit its applications in flame retardation of polymeric materials. Element doping is considered as a convenient and effective method for adjusting the properties of g-C_3_N_4_ [18]. Different elements, including boron, phosphorus, sulfur, and cobalt have been introduced into the g-C_3_N_4_ framework successfully [19,20]. It is widely accepted that phosphorus-containing additives contribute to enhancing the flame retardancy of polymers [21,22]. Furthermore, it has been proved that the presence of transition metal elements perform good catalytic oxidation of CO and organic volatiles during combustion of polymers [23]. Therefore, it is reasonable to expect that the doped g-C_3_N_4_ with phosphorus and transition metal elements may possess good flame retardant performance for polymers. To the best of our knowledge, the application of doped g-C_3_N_4_ in flame retardant polymer materials with outstanding efficiency is still not reported.

In this work, cobalt/phosphorus co-doped g-C_3_N_4_ (Co/P-C_3_N_4_) was prepared by a simple and scalable thermal decomposition method. Then the effects of the Co/P-C_3_N_4_ concentration on thermal stability, fire safety and mechanical properties of PLA composites were studied. Meanwhile, the mechanism for fire hazard reduction of PLA was also proposed. 

## 2. Materials and Methods

### 2.1. Materials

Polylactide (PLA, Ingeo 4032D) was supplied by Natureworks (Minnetonka, MN, USA). Hexachlorotriphosphazene (HCCP), melamine, and cobalt chloride (CoCl_2_) were purchased from J&K (Shanghai, China).

### 2.2. Preparation of Pure and Doped Graphitic Carbon Nitride

**Scheme 1:**
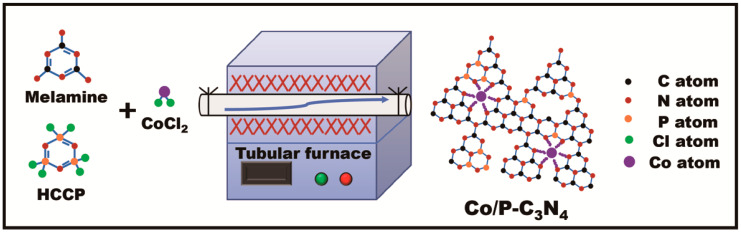
The synthesis route of Co/P-C_3_N_4_.

The pure and cobalt/phosphorus co-doped g-C_3_N_4_ (Co/P-C_3_N_4_) was prepared by one-step thermal decomposition method according to published work [24]. For pure graphitic carbon nitride (g-C_3_N_4_), the melamine was sealed a covered crucible and transformed into the tube furnace (OTF-1200X, Kejing Materials Technology Co, Hefei, China). A specimen was heated to 550 °C at a heating rate of 5 °C/min in air atmosphere, and maintained at 550 °C for 4 h to complete the reaction. For phosphorus and cobalt co-doped graphitic carbon nitride (Co/P-C_3_N_4_), the CoCl_2_·6H_2_O was first mixed with melamine in the weight ratio of 5/90, and dried in an air-circulating oven before grounding. Then, the HCCP was uniformly grounded and mixed with melamine and CoCl_2_. The weight ratio of HCCP and melamine was fixed at 1:9. Finally the sample was calcined as the same heating procedures. The synthesis diagram was shown in Scheme 1.

### 2.3. Preparation of PLA-Based Composites

A desired amount of the obtained g-C_3_N_4_ or Co/P-C_3_N_4_ was mixed with PLA in an internal mixer (Plastic-Corder, Brabender, Duisburg, Germany). The samples were processed by setting at 190 °C, 60 rpm for 8 min. Then, pure PLA and PLA composites were molded into standard specimens for further characterization by hot-compression (190 °C, 5 min). The samples were abbreviated as g-C_3_N_4_-X and Co/P-C_3_N_4_-X, respectively, where X stands for the weight ratio of flame retardant fillers in PLA composites. The formulations were listed in Table 1. 

### 2.4. Characterization

The morphology of the Co/P-C_3_N_4_ was observed by field-emission scanning electron microscopy (FE-SEM, Quata 250, FEI, Waltham, MA, USA) with an energy-dispersive X-ray spectroscopy (EDX, Oxford Instrument, Abington, UK). Wide angle x-ray diffraction (XRD) patterns of the samples were recorded by using a X-Ray diffractometer (SmartLab, Rigaku, Tokyo, Japan) in the 2θ range of 10°–80° with a step of 0.02°. X-ray photoelectron spectroscopy (XPS, 5802 spectrometer, Physical Electronics PHI, Kanagawa, Japan) was used to determine the elemental compositions and chemical state of different elements. The melting enthalpies and recrystallization enthalpies were traced by a differential scanning calorimetry (DSC, DSC-204F1, Netzsch, Selb, Germany). Samples were heated and cooled in temperatures ranging from 30 to 190 °C at a ramp rate of 10 °C/min under nitrogen flow. Thermogravimetric analysis (TGA) was carried out on a thermal gravimetric analyzer (TGA-209F3, Netzsch, Selb, Germany) from 30 to 700 °C at a ramp rate of 10 °C/min under nitrogen flow. Limited oxygen index (LOI) testing was conducted on an oxygen index instrument (HC-2, Jiangning Analytical Instrument, Nanjing, China). The dimensions of the specimens were 100 × 6.5 × 3.0 mm^3^. The vertical burning test was carried out on a self-designed UL-94 vertical flame chamber according to GB/T2408-2008 with sample sizes measuring 130 × 13 × 3.0 mm^3^. Flammability properties were measured by a cone calorimeter (Fire Testing Technology, West Sussex, UK) according to the standard of ISO 5660. Samples (100 × 100 × 3 mm^3^) were tested under a heat flux of 35 kW/m^2^. 

## 3. Results and Discussion

### 3.1. Characterization of Co/P-C_3_N_4_

**Figure 1:**
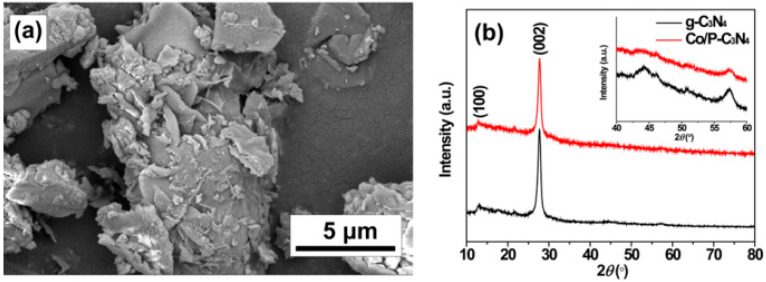
(**a**) SEM image of Co/P-C_3_N_4_, and (**b**) XRD patterns of g-C_3_N_4_ and Co/P-C_3_N_4_.

Figure 1a. shows the SEM image of the as-synthesized Co/P-C_3_N_4_. It is noted that the Co/P-C_3_N_4_ exhibits an aggregated, rough, and plate-like particles consisting of lamellar structures. The XRD patterns of the g-C_3_N_4_ and Co/P-C_3_N_4_ are presented in Figure 1b. It shows a strong diffraction peak at 27.7°, which is assigned to the (002) interplane of g-C_3_N_4_ induced by stacking of conjugated aromatic structures of thiotriazinone [25]. In addition, the peak located at 13.2° is ascribed to the diffraction of (100) plane. With the doping of cobalt (Co) and phosphorus (P) elements, the intensity of the diffraction peak at 43.1° and 57.4° decreases, suggesting that Co and P atoms have been doped into the structure of g-C_3_N_4_ successfully. To compare the thermal stability of g-C_3_N_4_ and Co/P-C_3_N_4_, TGA was performed under nitrogen atmosphere, as shown in Figure S1. It is obvious that the decomposition curve of Co/P-C_3_N_4_ shifts to a higher temperature, as compared with that of pure g-C_3_N_4_. Moreover, the residue at 800 °C for Co/P-C_3_N_4_ (6.68 wt %) is also higher than that of pure g-C_3_N_4_ (0.83 wt %). These results indicate that Co/P-C_3_N_4_ has a better thermal stability.

**Figure 2:**
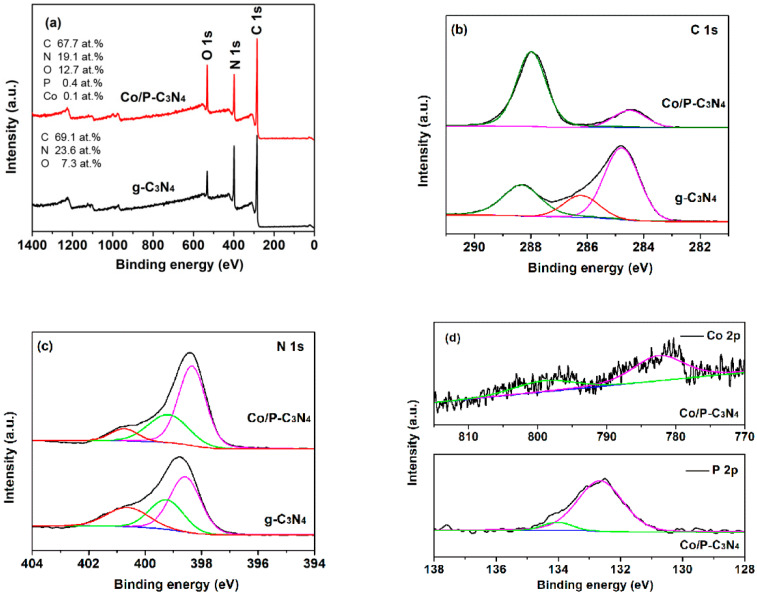
(**a**) XPS survey spectra, (**b**) C1s, (**c**) N1s, (**d**) P 2p and Co 2p of Co/P-C_3_N_4._

Figure 2a shows the XPS full survey spectra of g-C_3_N_4_ and Co/P-C_3_N_4_. It can be seen that there are three main peaks in the spectra, corresponding to C 1s, N 1s, and O 1s, respectively. The atomic percentage of carbon and nitrogen elements in Co/P-C_3_N_4_ decrease to 67.7 at.%, and 19.1 at.%, respectively, due to the introduction of cobalt and phosphorus. The presence of oxygen in g-C_3_N_4_ is due to the surface absorption and oxidation in air atmosphere, which is in good consistence with the reported results [24]. In addition, the oxygen content in Co/P-C_3_N_4_ increases significantly, compared with that of g-C_3_N_4_. It is conjectured that the Co/P element reacted with oxygen (O_2_) in air atmosphere during the copolymerization reaction. The high resolution C 1s spectrum of g-C_3_N_4_ in Figure 2b shows three obvious peaks at 284.8, 286.2, and 288.1 eV, which are attributed to graphitic carbon (C–C), carbon in C–NH_2_ species, and carbon in the N-containing aromatic ring (N–C=N), respectively [26,27]. With the doping of cobalt and phosphorus atoms, the N–C=N peak intensity for Co/P-C_3_N_4_ grows significantly, while the C–C peak intensity decreases simultaneously. In addition, the peak attributed to C-NH_2_ bond disappears, indicating that the presence of Co and P atoms contributes to the polymerization of graphitic carbon nitride. The high resolution N 1s spectrum of g-C_3_N_4_ in Figure 2c can be deconvoluted into three peaks with the binding energy of 398.6, 399.2 and 400.6 eV. The main peak at 398.6 eV is attributed to the aromatic nitrogen in triazine rings (C–N=C), while two weak peaks located at 399.4 eV and 400.7 eV refer to the sp^2^-hybridized N bonds, i.e., [C–N(–C)–C) and C–N(–H)–C] [28,29]. Moreover, the N 1s spectrum of Co/P-C_3_N_4_ exhibits three main deconvolution peaks at 398.4, 399.2 and 400.7 eV, showing a slight change after doping. The deconvoluted results of the N 1s spectra are shown in Table S1. It is observed that the atomic percentage of C–N=C groups for Co/P-C_3_N_4_ increases from 49.8% to 61.8%. As shown in Figure 2d, the signal peak centered at 132.7 eV in high resolution P 2p spectrum is ascribed to the P–N bond, while a weak band at 134.0 eV refers to the P=O bond, which further confirm that the doped phosphorous atoms most likely substitute the carbon atoms in Co/P-C_3_N_4_ [30,31]. The characteristic peaks at 782.5 eV and 799.0 eV for Co 2p spectrum in Figure 2d correspond to Co 2p_3/2_ and Co 2p_1/2_, respectively [32]. The above XPS results confirm the presence of Co and P elements, indicating the successful preparation of Co/P-C_3_N_4_.

### 3.2. Morphology of PLA Composites

**Figure 3:**
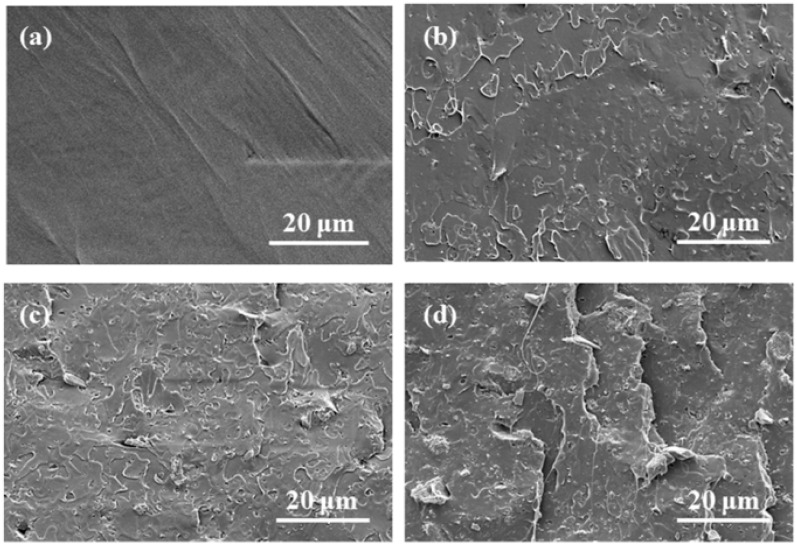
SEM images of pure PLA and PLA composites. (**a**) pure PLA, (**b**) Co/P-C_3_N_4_-2%, (**c**) Co/P-C_3_N_4_-5%, and (**d**) Co/P-C_3_N_4_-10%.

Figure 3 presents the SEM images of the fractured surfaces for PLA composites. It is noted that pure PLA in Figure 3a has a smooth and no-crinkled surface, which is corresponding to the brittle fracture [33]. The fractured surface of PLA composites containing Co/P-C_3_N_4_ (Figure 3b–d) becomes rougher with increasing Co/P-C_3_N_4_. Furthermore, many tiny cavities could be found on the surfaces in Figure 3c,d, indicating that the poor interfacial interaction between Co/P-C_3_N_4_ and PLA matrix with a loading over a critical value. 

### 3.3. Crystallization Behavior of PLA Composites

**Figure 4:**
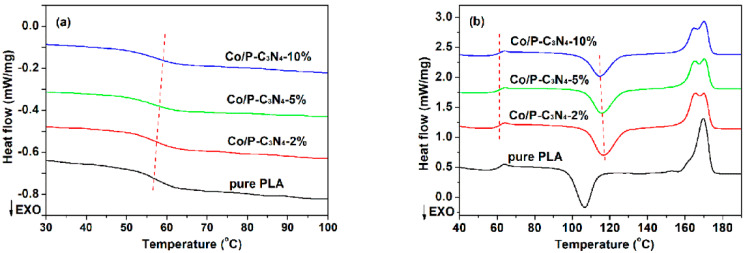
DSC curves of pure PLA and PLA composites. (**a**) First cooling, (**b**) second heating.

The crystallization behavior of PLA composites was measured by DSC. Figure 4 depicts the first cooling and second heating curves of PLA composites as a function of Co/P-C_3_N_4_ content. As shown in Figure 4a, no crystallization peaks appear during the cooling process, which is caused by the rigid segments in the PLA main chain [34]. As shown in Table 2, the glass transition temperature (*T*_g_) of the PLA composites are higher than that of pure PLA, which suggests that the introduction of Co/P-C_3_N_4_ hinders the movement of the PLA segments. The exothermic peaks observed between 100 and 130 °C in Figure 4b are associated with the cold crystallization of PLA. In addition, it is noted that double endothermic peaks appear around 170 °C in the Co/P-C_3_N_4_ composites, which are related to the melting of PLA. Fukushima et al. reported that the melt-recrystallization phenomenon of PLA was ascribed to different forms of crystals [35]. The low-melting peak (*T*_m1_) relates to the *β*-crystal, which is an imperfect crystal structure, while the high-melting peak (*T*_m2_) is assigned to the most common polymorph *α*-crystal of PLA. This result indicates that the addition of Co/P-C_3_N_4_ will restrict the mobility of PLA chains, which is similar to other PLA-based composites [36]. The cold-crystallization enthalpy (Δ*H*_cc_) of Co/P-C_3_N_4_-2% shows a little increase as compared with that of pure PLA. With further increasing the content of Co/P-C_3_N_4_, Δ*H*_cc_ decreases significantly. Similarly, the melting enthalpies (Δ*H*_m_) exhibit a decrease trend with the filler content. In addition, the crystallinity degree (*χ*_c_) of PLA composites is lower than that of pure PLA, indicating that Co/P-C_3_N_4_ has no nucleation effect on PLA matrix [37]. 

### 3.4. Thermal Stability of PLA Composites

**Figure 5:**
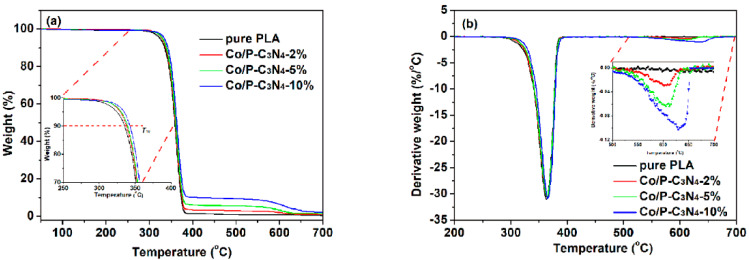
(**a**) TGA, and (**b**) DTG curves of PLA composites under nitrogen atmosphere.

The thermal stability of PLA composites under nitrogen atmosphere were examined with the detailed results shown in Figure 5 and Table 3. *T*_10_ represents the initial decomposition temperature where 10% weight loss occurs, while *T*_max_ refers to the temperature at the maximum mass loss rate. As shown in Figure 5a, the *T*_10_ values are 338.1, 339.8 and 342.8 °C for Co/P-C_3_N_4_-2%, Co/P-C_3_N_4_-5% and Co/P-C_3_N_4_-10%, respectively, which are all slightly higher than that of pure PLA (335.1 °C). This phenomenon may be ascribed to the labyrinth effect of Co/P-C_3_N_4_ in the composites [38]. From Figure 5b, it is observed that pure PLA shows only one peak between 300 and 380 °C, which is ascribed to the chain scission of PLA. Two decomposition peaks are observed for PLA composites. The main peak in the temperature range of 300–400 °C is similar to pure PLA, while the small peak between 500 and 650 °C corresponds to the decomposition of Co/P-C_3_N_4_. This is due to the thermal decomposition of Co/P-C_3_N_4_ at a higher temperature. As shown in Table 3, pure PLA shows almost no char residues at 700 °C. whereas the char residues of PLA composites increase continuously with increasing Co/P-C_3_N_4_ content, which are attributed to the catalytic charring capability of Co/P-C_3_N_4_.

### 3.5. Fire Performance of PLA Composites

LOI and UL-94 vertical burning tests are widely utilized to assess the flame retardant performance of polymers for specific engineering applications. The LOI and UL-94 test results for pure PLA and its composites are presented in Table 1. Pure PLA has a lower LOI value of 19.5% and no rating in UL-94 test, indicating it is highly susceptible to flames. The LOI values of PLA composites exhibit a slightly increase with the addition of Co/P-C_3_N_4_. Moreover, the presence of Co/P-C_3_N_4_ had a positive effect on the UL-94 vertical burning tests. The samples of PLA composite containing 10 wt % Co/P-C_3_N_4_ exhibit a reduced burning rate and pass V-1 rating, which is much better than those of pure PLA. However, the dripping phenomenon of PLA is still persisted.

**Figure 6:**
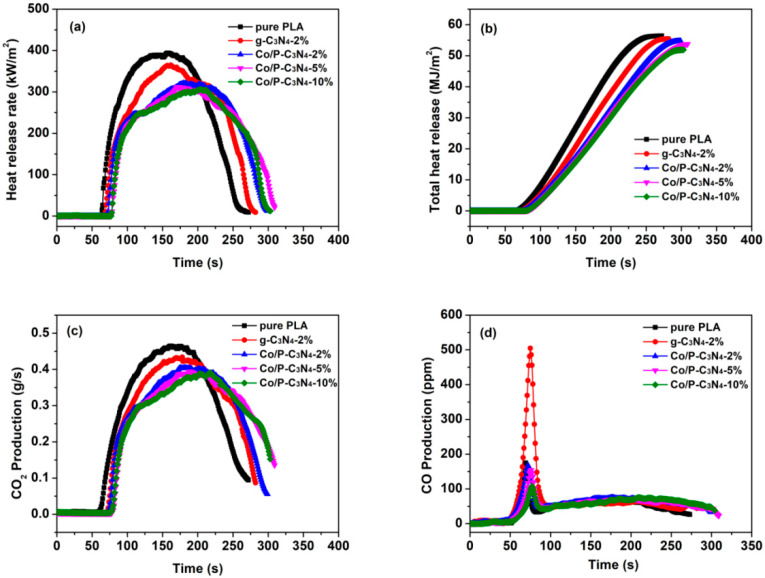
Cone calorimeter test results of pure PLA and PLA composites: (**a**) heat release rates, (**b**) total heat release, (**c**) CO_2_ production rate, and (**d**) CO production rate as a function of time.

Cone calorimetry is one of the most comprehensive methods to measure the combustion performance of polymer materials [39,40,41]. The heat release rate (HRR), total heat release (THR), carbon dioxide (CO_2_) production rate (CO2P) and carbon monoxide (CO) production rate (COP) curves of pure PLA and its composites are shown in Figure 6, and the corresponding data are listed in Table 4. In Figure 6a, the peak of HRR (pHRR) of g-C_3_N_4_-2% reduces to 364.8 kW/m^2^ showing a slight decrease, compared with that of pure PLA (394.3 kW/m^2^). This is due to the fact that g-C_3_N_4_ serves as a thermal barrier to reduce rapid heat release during combustion [42]. In the case of Co/P-C_3_N_4_-2%, the pHRR value decreases to 321.0 kW/m^2^, indicating that Co/P-C_3_N_4_ exhibits a better barrier effect than g-C_3_N_4_. Moreover, a significant delay in time to ignition (TTI) is observed for PLA-Co/P-C_3_N_4_ composites. Particularly, the maximum delay is observed for Co/P-C_3_N_4_-10% (~79 s). The THR curves of pure PLA and PLA composites are presented in Figure 6b. It is evident that the THR of PLA composites containing Co/P-C_3_N_4_ decreases continuously with the increase of Co/P-C_3_N_4_ content. For instance, the THR value of Co/P-C_3_N_4_-10% decreases from 56.4 to 51.9 MJ/m^2^. Toxic gases and fumes are regarded as larger threatens during fire accident [43]. The CO_2_ production rate (CO_2_P) curves of pure PLA and its composites are depicted in Figure 6c. It is observed that the CO_2_P exhibits a similar tendency to HRR value. The peak CO_2_ production rate (PCO_2_P) for Co/P-C_3_N_4_-10% decreases to 0.390 g/s, which is reduced by 16.2%, as compared to that of pure PLA. It is worthy to note that the peak of CO production rate (PCOP) of g-C_3_N_4_-2% increases up to 504.7 ppm (Figure 6d). It is speculated that the introduction of pure g-C_3_N_4_ isolates oxygen, resulting in the incomplete combustion of PLA matrix. In comparison, the presence of Co/P-C_3_N_4_ can inhibit the production rate of CO. With the incorporation of 10 wt % Co/P-C_3_N_4_, the PCOP value reduces noticeably by 38.5% compared to pure PLA (174.9 ppm). Co atoms can play a catalytic role during combustion and convert toxic gases such as CO into non-toxic gases, which is very conducive to evacuation in a fire [44,45].

### 3.6. Analysis of Char Residues

**Figure 7:**
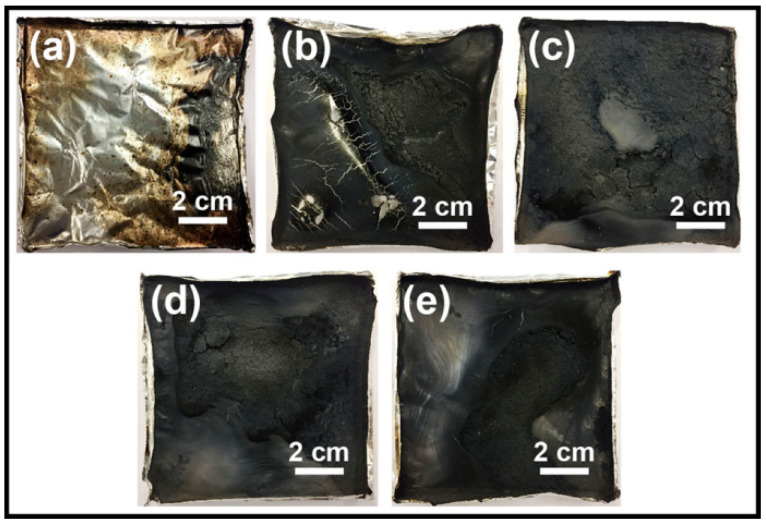
Digital photos of char residues after cone calorimeter tests: (**a**) pure PLA, (**b**) g-C_3_N_4_-2%, (**c**) Co/P-C_3_N_4_-2%, (**d**) Co/P-C_3_N_4_-5%, and (**e**) Co/P-C_3_N_4_-10%.

**Figure 8:**
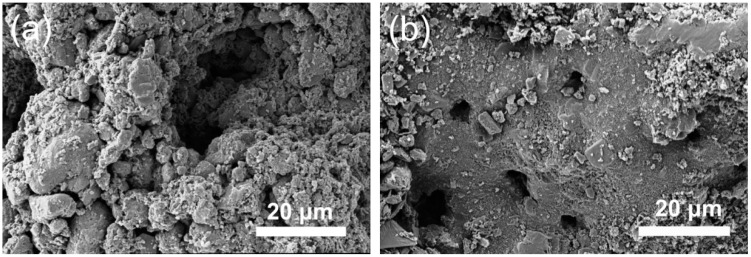
SEM images of char residues (**a**) g-C_3_N_4_-2%, and (**b**) Co/P-C_3_N_4_-2%.

Digital photos of char residues are repented in Figure 7. Almost no char residues can be observed for control PLA, as shown in Figure 7a, indicating that pure PLA is completely burnt out. With the addition of 2 wt % g-C_3_N_4_, the char residues exhibit many cracks and unintegrated structure (Figure 7b). Meanwhile, the PLA composites containing Co/P-C_3_N_4_ (Figure 7c–e) have thick, integrated, and dense carbonization zone. With increasing the Co/P-C_3_N_4_ content, the char layer becomes thicker which is favorable for preventing heat conduction and gas diffusion to inhibit the combustion [46]. To observe the micromorphology of the char residues, the SEM images are presented in Figure 8. Figure 8a shows that the char residues of g-C_3_N_4_-2% exhibit a loose and porous structure with a large number of honeycomb piles-like pores, which cannot hinder the burning efficiently. For Co/P-C_3_N_4_-2% (Figure 8b), the compact and continuous char layer can be observed, suggesting that the char-forming process is improved. The presence of Co and P elements in doped g-C_3_N_4_ contributes to the formation of intact char residues to hinder heat transfer and slow down the diffusion of gas during combustion. 

**Figure 9:**
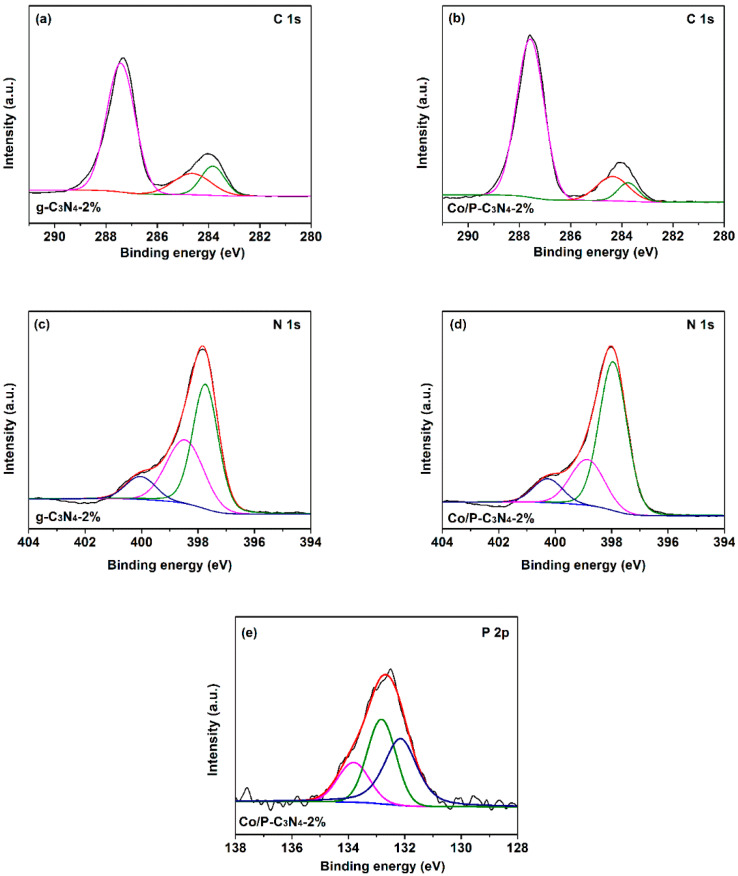
High resolution XPS spectra of char residues. (**a**) C 1s, and (**c**) N 1s, of g-C_3_N_4_, (**b**) C 1s, (**d**) N 1s, and (**e**) P 2p, of Co/P-C_3_N_4_.

The chemical composition of char residues was examined by XPS, and the relevant results are shown in Figure 9 and Table S2. It is observed that the high solution C 1s spectrum of g-C_3_N_4_-2% in Figure 9a can be deconvoluted into three peaks at 287.4, 284.8 and 283.9eV, corresponding to C=O, C–C and graphitic C–C, respectively. As shown in Figure 9b, the deconvoluted peaks at 287.6, 284.4 and 283.8 eV for C 1s spectrum are assigned to C=O/C–P, C–C and graphitic C–C. As observed in Table S2, the atomic percentage of graphitic C in the char residues of Co/P-C_3_N_4_-2% decreases from 17.8% to 7.1% for g-C_3_N_4_-2%. Meanwhile, the atomic ratio of C=O and/or C-P bonds increases from 70.4% to 79%. The N 1s spectra (Figure 9c,d) of the char residues for g-C_3_N_4_-2% and Co/P-C_3_N_4_-2% are fitted to three similar peaks. The percentage of O–N=O bonds (397.9 eV) in the char residue of Co/P-C_3_N_4_-2% is 65.7%, which is higher than that of g-C_3_N_4_-2% (53.2%). Moreover, the high-resolution spectrum of P 2p in Figure 9e indicates the coexistence of P–C (133.8 eV), O–P=O (132.8 eV) and P–O–P (132.1 eV). Such char layer is believed to serve as a better physical barrier in protecting the underlying polymers against a flame [47,48,49]. 

**Figure 10:**
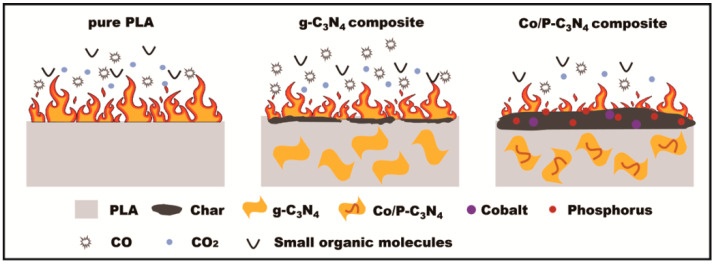
Schematic diagram of flame retardant mechanism of PLA composites.

Based on the abovementioned analysis, the possible flame retardant mechanism is illustrated in Figure 10. Pure PLA absorbs heat and releases volatile gaseous without any restriction upon exposure to a fire. It will release a large amount of smoke and toxic gases (CO) due to continuous fuel supply, which will increase the potential fire hazard of PLA. With the addition of g-C_3_N_4_, PLA composites can form a loose and discontinuous char layer with many cracks. The heat and oxygen can penetrate into this char layer easily, resulting in a low protection efficiency for underlying polymers [50]. Noticeably, the CO production rate of PLA/g-C_3_N_4_ composite increases significantly as compared to that of pure PLA. It is conjectured that the char layer isolates oxygen and fuel, resulting in the incomplete combustion of PLA. As a comparison, the char residue of PLA composite containing Co/P-C_3_N_4_ shows a compact and coherent structure. In addition, P/Co co-doped -C_3_N_4_ generates more active sites with high catalytic capability in addition to catalytic role of Coin char layer, which contributes to oxidation of the toxic pyrolysis product, i.e., CO into CO_2_ [51]. 

### 3.7. Mechanical Properties

**Figure 11:**
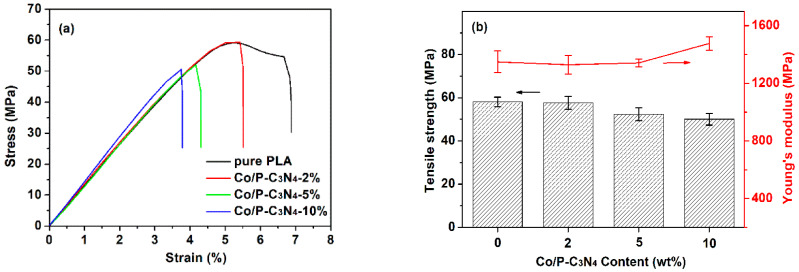
(**a**) Typical stress-strain curves, (**b**) tensile properties of pure PLA and PLA composites.

The tensile stress-strain curves of PLA composites are shown in Figure 11a. It is observed that both the tensile strength and elongation at the break of the PLA composites decrease continuously as compared with those of pure PLA. The higher loading of the Co/P-C_3_N_4_ results in larger Young’s modulus and smaller elongation at break. It can be explained that the Co/P-C_3_N_4_ serves as a rigid filler to restrict the movement of PLA molecular chains and bear the force themselves [52,53]. However, the poor interface adhesion between Co/P-C_3_N_4_ and PLA, as shown in Figure 3, will lead to a decrease in tensile strength. Figure 11b presents the dependence of tensile strength and Young’s modulus of PLA composites on Co/P-C_3_N_4_ content. It is found that pure PLA belongs to a brittle material with a tensile strength of 58.05 MPa and a Young’s modulus of 1349 MPa. With the addition of 10 wt % Co/P-C_3_N_4_, the tensile strength of PLA composites decreases to 50.21 MPa. Meanwhile, the Young’s modulus of Co/P-C_3_N_4_-10% reaches 1496 MPa, a 10.9% improvement, compared with that of pure PLA.

## 4. Conclusions

In this work, we demonstrated a facile strategy to prepare Co/P-C_3_N_4_ by a simple and scalable thermal decomposition method, which was then utilized to prepare flame retardant PLA composites. The structure of Co/P-C_3_N_4_ was confirmed by SEM, XRD and XPS. Then, the effects of Co/P-C_3_N_4_ concentration on crystallization behavior, thermal stability, fire performance and mechanical properties of PLA were investigated. The results revealed that Co/P-C_3_N_4_ could restrict the mobility of PLA chains to some extent. The thermal stability of PLA composites was increased continuously with increasing the content of Co/P-C_3_N_4_. The PLA composite containing 10 wt % Co/P-C_3_N_4_ exhibited better fire resistance with a moderate decrease in PHRR, PCO2P and PCOP values, by 22.4%, 16.2%, and 38.5%, respectively. In addition, the analysis of char residues revealed that the addition of Co/P-C_3_N_4_ contributed to the formation of protective char layer with compact and continuous structure. These improvements in thermal stability and fire retardation of PLA can be ascribed to the physical barrier effect, as well as catalytic effects of Co/P-C_3_N_4_. Furthermore, the introduction of Co/P-C_3_N_4_ resulted in a slight decrease in tensile strength as compared to pure PLA. 

## Figures and Tables

**Table 1:** Formulations, LOI and UL-94 test results of PLA composites.

Samples	PLA (wt %)	g-C_3_N_4_ (wt %)	Co/P-C_3_N_4_ (wt %)	LOI (%)	UL-94 Test			
					t_1_ (s)	t_2_ (s)	UL-94 Rate	Dripping
Pure PLA	100	--	--	19.5	>50	--	NR	Yes
g-C_3_N_4_-2%	98	2	--	20	21.3	10.2	V-2	Yes
Co/P-C_3_N_4_-2%	98	--	2	21	15.8	20.4	V-2	Yes
Co/P-C_3_N_4_-5%	95	--	5	22	14.7	13.3	V-2	Yes
Co/P-C_3_N_4_-10%	90	--	10	22.5	12.8	3.5	V-1	Yes

**Table 2:** DSC data of PLA composites.

Samples	*T*_g_ (°C)	*T*_cc_ (°C)	Δ*H*_cc_ (J/g)	*T*_m1_ (°C)	*T*_m2_ (°C)	Δ*H*_m_ (J/g)	*χ*_c_ (%)
Pure PLA	61.5	106.5	35.5	--	169.6	41.2	6.1
Co/P-C_3_N_4_-2%	62.1	116.5	36.0	165.6	169.7	38.9	3.2
Co/P-C_3_N_4_-5%	62.2	115.7	28.6	165.3	170.1	31.8	3.6
Co/P-C_3_N_4_-10%	62.3	114.7	27.3	165.1	170.0	31.3	4.6

**Table 3:** TGA data of PLA composites.

Samples	*T*_10_ (°C)	*T*_max_ (°C)	Char Residue at 700 °C (wt %)
Pure PLA	335.6	364.9	0.03
Co/P-C_3_N_4_-2%	338.1	365.2	1.65
Co/P-C_3_N_4_-5%	339.8	364.9	1.78
Co/P-C_3_N_4_-10%	342.8	365.8	1.96

**Table 4:** Combustion results of control PLA and its composites obtained from cone calorimeter tests.

Samples	TTI (s)	*T*_PHRR_ (s)	PHRR (kW/m^2^)	THR (MJ/m^2^)	Char Residue (wt %)	PCO_2_P (g/s)	PCOP (ppm)
Pure PLA	63	158	394.3	56.4	0.1	0.465	174.9
g-C_3_N_4_-2%	69	162	364.8	55.4	3.9	0.434	504.7
Co/P-C_3_N_4_-2%	73	180	321.0	54.7	5.5	0.405	168.0
Co/P-C_3_N_4_-5%	78	176	311.9	53.8	7.1	0.394	154.3
Co/P-C_3_N_4_-10%	79	204	306.1	51.9	8.9	0.390	107.6

## Supplementary Materials

The following are available online at https://www.mdpi.com/2073-4360/12/5/1106/s1, Figure S1: TGA curves of g-C_3_N_4_ and Co-C_3_N_4_ at a heating rate of 10 °C/min under nitrogen atmosphere. Table S1: Deconvoluted results of the XPS N 1s spectra of g-C_3_N_4_ and Co/P-C_3_N_4_. Table S2: XPS Fitting results of char residues.

## References

[1] Vink E.T.H., Rabago K.R., Glassner D.A., Springs B., O’Connor R.P., Kolstad J., Gruber P.R. (2004). The sustainability of NatureWorks (TM) polylactide polymers and Ingeo (TM) polylactide fibers(a): An update of the future. Macromol. Biosci..

[2] Wu W., Han S.T., Venkatesh S., Sun Q.J., Peng H.Y., Zhou Y., Yeung C.C., Li R.K.Y., Roy V.A.L. (2018). Biodegradable skin-Inspired nonvolatile resistive switching memory based on gold nanoparticles embedded alkali lignin. Org. Electron..

[3] Tawiah B., Yu B., Wei R.C., Yuen R.K.K., Chen W., Xin J.H., Fei B. (2019). Simultaneous fire safety enhancement and mechanical reinforcement of poly (lactic acid) biocomposites with hexaphenyl (nitrilotris(ethane-2,1-diyl))tris (phosphoramidate). J. Hazard. Mater..

[4] Mayekar P.C., Castro-Aguirre E., Auras R., Selke S., Narayan R. (2020). Effect of nano-Clay and surfactant on the biodegradation of poly(lactic acid) Films. Polymers.

[5] Tarres Q., Oliver-Ortega H., Espinach F.X., Mutje P., Delgado-Aguilar M., Mendez J.A. (2019). Determination of mean intrinsic flexural strength and coupling factor of natural fiber reinforcement in polylactic acid biocomposites. Polymers.

[6] Jing J., Zhang Y., Fang Z.P., Wang D.Y. (2018). Core-Shell flame retardant/graphene oxide hybrid: A self-Assembly strategy towards reducing fire hazard and improving toughness of polylactic acid. Compos. Sci. Technol..

[7] Cheng K.C., Yu C.B., Guo W.J., Wang S.F., Chuang T.H., Lin Y.H. (2012). Thermal properties and flammability of polylactide nanocomposites with aluminum trihydrate and organoclay. Carbohyd. Polym..

[8] Zhang S., Yan Y.X., Wang W.J., Gu X.Y., Li H.F., Li J.H., Sun J. (2018). Intercalation of phosphotungstic acid into layered double hydroxides by reconstruction method and its application in intumescent flame retardant poly (lactic acid) composites. Polym. Degrad. Stabil..

[9] Tawiah B., Yu B., Fei B. (2018). Advances in Flame Retardant Poly(Lactic Acid). Polymers.

[10] Liu J., Wang H., Antonietti M. (2016). Graphitic carbon nitride “reloaded”: Emerging applications beyond (photo) catalysis. Chem. Soc. Rev..

[11] Lau V.W.H., Moudrakovski I., Botari T., Weinberger S., Mesch M.B., Duppel V., Senker J., Blum V., Lotsch B.V. (2016). Rational design of carbon nitride photocatalysts by identification of cyanamide defects as catalytically relevant sites. Nat. Commun..

[12] Chen Z., Yu X., Zhu Q.H., Fan T.T., Wu Q.L., Zhang L.Z., Li J.H., Fang W.P., Yi X.D. (2018). Steam engraving optimization of graphitic carbon nitride with enhanced photocatalytic hydrogen evolution. Carbon.

[13] Cheng J.S., Hu Z., Lv K.L., Wu X.F., Li Q., Li Y.H., Li X.F., Sun J. (2018). Drastic promoting the visible photoreactivity of layered carbon nitride by polymerization of dicyandiamide at high pressure. Appl. Catal. B Environ..

[14] Shi Y.Q., Wang L.C., Fu L.B., Liu C., Yu B., Yang F.Q., Hu Y. (2019). Sodium alginate-Templated synthesis of g-C3N4/carbon spheres/Cu ternary nanohybrids for fire safety application. J. Colloid Interf. Sci..

[15] Shi Y.Q., Long Z., Yu B., Zhou K.Q., Gui Z., Yuen R.K.K., Hu Y. (2015). Tunable thermal, flame retardant and toxic effluent suppression properties of polystyrene based on alternating graphitic carbon nitride and multi-walled carbon nanotubes. J. Mater. Chem. A.

[16] Shi Y.Q., Liu C., Fu L.B., Yang F.Q., Lv Y.C., Yu B. (2019). Hierarchical assembly of polystyrene/graphitic carbon nitride/reduced graphene oxide nanocomposites toward high fire safety. Compos. Part B Eng..

[17] Shi Y.Q., Yu B., Duan L.J., Gui Z., Wang B.B., Hu Y., Yuen R.K.K. (2017). Graphitic carbon nitride/phosphorus-rich aluminum phosphinates hybrids as smoke suppressants and flame retardants for polystyrene. J. Hazard. Mater..

[18] Jiang L.B., Yuan X.Z., Pan Y., Liang J., Zeng G.M., Wu Z.B., Wang H. (2017). Doping of graphitic carbon nitride for photocatalysis: A review. Appl. Catal. B Environ..

[19] Chen D.D., Liu J.G., Jia Z.Z., Fang J.Z., Yang F., Tang Y.M., Wu K., Liu Z., Fang Z.Q. (2019). Efficient visible-Light-Driven hydrogen evolution and Cr(VI) reduction over porous P and Mo co-doped g-C3N4 with feeble N vacancies photocatalyst. J. Hazard. Mater..

[20] Wang K.Y., Gu G.Z., Hu S.Z., Zhang J., Sun X.L., Wang F., Li P., Zhao Y.F., Fan Z.P., Zou X. (2019). Molten salt assistant synthesis of three-Dimensional cobalt doped graphitic carbon nitride for photocatalytic N-2 fixation: Experiment and DFT simulation analysis. Chem. Eng. J..

[21] Rao W.H., Liao W., Wang H., Zhao H.B., Wang Y.Z. (2018). Flame-Retardant and smoke-Suppressant flexible polyurethane foams based on reactive phosphorus-containing polyol and expandable graphite. J. Hazard. Mater..

[22] Shi Y.Q., Yu B., Zheng Y.Y., Yang J., Duan Z.P., Hu Y. (2018). Design of reduced graphene oxide decorated with DOPO-phosphanomidate for enhanced fire safety of epoxy resin. J. Colloid Interf. Sci..

[23] Xu W.Z., Wang X.L., Wu Y., Li W., Chen C.Y. (2019). Functionalized graphene with Co-ZIF adsorbed borate ions as an effective flame retardant and smoke suppression agent for epoxy resin. J. Hazard. Mater..

[24] Li X.F., Zhang J., Shen L.H., Ma Y.M., Lei W.W., Cui Q.L., Zou G.T. (2009). Preparation and characterization of graphitic carbon nitride through pyrolysis of melamine. Appl. Phys. A Mater..

[25] Zhao Y.C., Yu D.L., Zhou H.W., Tian Y.J., Yanagisawa O. (2005). Turbostratic carbon nitride prepared by pyrolysis of melamine. J. Mater. Sci..

[26] Zhu Y.P., Ren T.Z., Yuan Z.Y. (2015). Mesoporous phosphorus-Doped g-C3N4 nanostructured flowers with superior photocatalytic hydrogen evolution performance. ACS Appl. Mater. Inter..

[27] Gu S., Xie J.L., Li C.M. (2014). Hierarchically porous graphitic carbon nitride: Large-Scale facile synthesis and its application toward photocatalytic dye degradation. RSC Adv..

[28] Zhou Y.J., Zhang L.X., Liu J.J., Fan X.Q., Wang B.Z., Wang M., Ren W.C., Wang J., Li M.L., Shi J.L. (2015). Brand new P-doped g-C3N4: Enhanced photocatalytic activity for H2 evolution and Rhodamine B degradation under visible light. J. Mater. Chem. A.

[29] Wang B., Yin X.H., Peng D., Lv R.H., Na B., Liu H.S., Gu X.B., Wu W., Zhou J.L., Zhang Y. (2020). Achieving thermally conductive low loss PVDF-based dielectric composites via surface functionalization and orientation of SiC nanowires. Express Polym. Lett..

[30] Zhang Y.J., Mori T., Ye J.H., Antonietti M. (2010). Phosphorus-Doped carbon nitride solid: Enhanced electrical conductivity and photocurrent generation. J. Am. Chem. Soc..

[31] Lan D.H., Wang H.T., Chen L., Au C.T., Yin S.F. (2016). Phosphorous-Modified bulk graphitic carbon nitride: Facile preparation and application as an acid-Base bifunctional and efficient catalyst for CO2 cycloaddition with epoxides. Carbon.

[32] Chen P.W., Li K., Yu Y.X., Zhang W.D. (2017). Cobalt-Doped graphitic carbon nitride photocatalysts with high activity for hydrogen evolution. Appl. Surf. Sci..

[33] Wu W., Cao X.W., Zhang Y.J., He G.J. (2013). Polylactide/halloysite nanotube nanocomposites: Thermal, mechanical properties, and foam processing. J. Appl. Polym. Sci..

[34] Wu W., Liu T., Zhang D.L., Sun Q.J., Cao K., Zha J.W., Lu Y., Wang B., Cao X.W., Feng Y.H. (2019). Significantly improved dielectric properties of polylactide nanocomposites via TiO2 decorated carbon nanotubes. Compos. Part A Appl. S..

[35] Fukushima K., Tabuani D., Arena M., Gennari M., Camino G. (2013). Effect of clay type and loading on thermal, mechanical properties and biodegradation of poly(lactic acid) nanocomposites. React. Funct. Polym..

[36] Orue A., Eceiza A., Arbelaiz A. (2018). The effect of sisal fiber surface treatments, plasticizer addition and annealing process on the crystallization and the thermo-Mechanical properties of poly(lactic acid) composites. Ind. Crop. Prod..

[37] Mngomezulu M.E., Luyt A.S., John M.J. (2019). Morphology, thermal and dynamic mechanical properties of poly (lactic acid)/expandable graphite (PLA/EG) flame retardant composites. J. Thermoplast. Compos..

[38] Shi Y.Q., Liu C., Liu L., Fu L.B., Yu B., Lv Y.C., Yang F.Q., Song P.A. (2019). Strengthening, toughing and thermally stable ultra-Thin MXene nanosheets/polypropylene nanocomposites via nanoconfinement. Chem. Eng. J..

[39] Lin B., Yuen A.C.Y., Li A., Zhang Y., Chen T.B.Y., Yu B., Lee E.W.M., Peng S.H., Yang W., Lu H.D. (2020). MXene/chitosan nanocoating for flexible polyurethane foam towards remarkable fire hazards reductions. J. Hazard. Mater..

[40] Yu B., Tawiah B., Wang L.Q., Yuen A.C.Y., Zhang Z.C., Shen L.L., Lin B., Fei B., Yang W., Li A. (2019). Interface decoration of exfoliated MXene ultra-thin nanosheets for fire and smoke suppressions of thermoplastic polyurethane elastomer. J. Hazard. Mater..

[41] Yu B., Xing W.Y., Guo W.W., Qiu S.L., Wang X., Lo S.M., Hu Y. (2016). Thermal exfoliation of hexagonal boron nitride for effective enhancements on thermal stability, flame retardancy and smoke suppression of epoxy resin nanocomposites via sol-gel process. J. Mater. Chem. A.

[42] Cao X.W., Chi X.N., Deng X.Q., Liu T., Yu B., Wang B., Yuen A.C.Y., Wu W., Li R.K.Y. (2020). Synergistic effect of flame retardants and graphitic carbon nitride on flame retardancy of polylactide composites. Polym. Advan. Technol..

[43] Wu W., He H.B., Liu T., Wei R.C., Cao X.W., Sun Q.J., Venkatesh S., Yuen R.K.K., Roy V.A.L., Li R.K.Y. (2018). Synergetic enhancement on flame retardancy by melamine phosphate modified lignin in rice husk ash filled P34HB biocomposites. Compos. Sci. Technol..

[44] Ye X.M., Zhang W.C., Yang R.J., He J.Y., Li J.R., Zhao F.Q. (2020). Facile synthesis of lithium containing polyhedral oligomeric phenyl silsesquioxane and its superior performance in transparency, smoke suppression and flame retardancy of epoxy resin. Compos. Sci. Technol..

[45] Yang W., Yang W.J., Tawiah B., Zhang Y., Wang L.L., Zhu S.E., Chen T.B.Y., Yuen A.C.Y., Yu B., Liu Y.F. (2018). Synthesis of anhydrous manganese hypophosphite microtubes for simultaneous flame retardant and mechanical enhancement on poly(lactic acid). Compos. Sci. Technol..

[46] Tawiah B., Yu B., Yuen A.C.Y., Yuen R.K.K., Xin J.H., Fei B. (2019). Thermal, crystalline and mechanical properties of flame retarded Poly (lactic acid) with a PBO-like small molecule-Phenylphosphonic Bis (2-aminobenzothiazole). Polym. Degrad. Stabil..

[47] Gong K.L., Zhou K.Q., Yu B. (2020). Superior thermal and fire safety performances of epoxy-based composites with phosphorus-doped cerium oxide nanosheets. Appl. Surf. Sci..

[48] Nine M.J., Tran D.N.H., Tung T.T., Kabiri S., Losic D. (2017). Graphene-Borate as an efficient fire retardant for cellulosic materials with multiple and synergetic modes of action. ACS Appl. Mater. Inter..

[49] Nine M.J., Tran D.N.H., ElMekawy A., Losic D. (2017). Interlayer growth of borates for highly adhesive graphene coatings with enhanced abrasion resistance, fire-retardant and antibacterial ability. Carbon.

[50] Yuan Y., Yu B., Shi Y.Q., Ma C., Song L., Hu W.Z., Hu Y. (2018). Highly efficient catalysts for reducing toxic gases generation change with temperature of rigid polyurethane foam nanocomposites: A comparative investigation. Compos. Part A Appl. S..

[51] Mu X.W., Pan Y., Ma C., Zhan J., Song L. (2018). Novel Co3O4/covalent organic frameworks nanohybrids for conferring enhanced flame retardancy, smoke and CO suppression and thermal stability to polypropylene. Mater. Chem. Phys..

[52] Ren Y.J., Zhang Y.F., Fang H.M., Ding T.P., Li J.L., Bai S.L. (2018). Simultaneous enhancement on thermal and mechanical properties of polypropylene composites filled with graphite platelets and graphene sheets. Compos. Part A Appl. S..

[53] Zhao H., She W., Shi D., Wu W., Zhang Q.C., Li R.K.Y. (2019). Polyurethane/POSS nanocomposites for superior hydrophobicity and high ductility. Compos. Part B Eng..

