# Supplementary material for: Facile Synthesis of Phosphorus and Cobalt Co-Doped Graphitic Carbon Nitride for Fire and Smoke Suppressions of Polylactide Composite
Source: Polymers (Basel). 2020 May 12;12(5):1106. doi: 10.3390/polym12051106 (PMC7285335; doi:10.3390/polym12051106)
Supplement: Supplementary file 1 [file polymers-12-01106-s001.pdf]

Supporting Information

Facile Synthesis of Phosphorus and Cobalt Co-Doped Graphitic Carbon Nitride for Fire and Smoke Suppressions of Polylactide Composite

Xianwu Cao [1], Xiaoning Chi [1], Xueqin Deng [1], Qijun Sun [2], Xianjing Gong [2], Bin Yu [3,*], Anthony Chun Yin Yuen [4], Wei Wu [1,2,*] and Robert Kwow Yiu Li [2]

[1] National Engineering Research Center of Novel Equipment for Polymer Processing, Key Laboratory of Polymer Processing Engineering of Ministry of Education, Guangdong Provincial Key Laboratory of Technique and Equipment for Macromolecular Advanced Manufacturing, South China University of Technology, Guangzhou 510640, China; ppeme@scut.edu.cn (X.C.); cxnnxc0402@163.com (X.C.); xueqindd@163.com (X.D.)

[2] Department of Materials Science and Engineering, City University of Hong Kong, Tat Chee Avenue, Kowloon, Hong Kong SAR 999077, China; qjsun2-c@my.cityu.edu.hk (Q.S.); jingxgong2-c@my.cityu.edu.hk (X.G.); aprkyl@city.edu.hk (R.K.Y.L.)

[3] Centre for Future Materials, University of Southern Queensland, Toowoomba 4350, Australia

[4] School of Mechanical and Manufacturing Engineering, University of New South Wales, Sydney 2052, Australia; c.y.yuen@unsw.edu.au

* Correspondence: bin.yu@usq.edu.au (B.Y.); weiwu39@scut.edu.cn (W.W.)

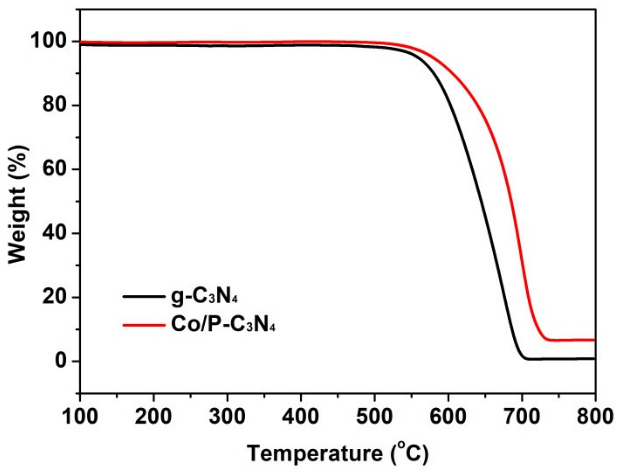

Figure S1. TGA curves of g-C_3N_4 and Co-C_3N_4 at a heating rate of 10 ºC/min under nitrogen atmosphere.

Table S1 Deconvoluted results of the XPS N 1s spectra of g-C_3N_4 and Co/P-C_3N_4.

Samples	Peaks	Binding energy (eV)	Area	Atomic percentage (%)	FWHM(eV)
	1	398.6	2531.9	49.8	1.285
g-C_3N_4	2	399.2	1423.9	28.0	1.401
	3	400.6	1127.2	22.2	1.707
	1	398.3	8531.7	61.8	1.134
Co/P-C_3N_4	2	399.4	4080.3	29.6	1.656
	3	400.7	1192.1	8.6	1.118

Table S2. XPS Fitting results of char residues.

Samples	Elements	Peaks	Binding energy (eV)	Atomic percentage (%)	FWHM(eV)
g-C_3N_4-2%	C 1s	1	283.9	17.8	1.158
		2	284.8	11.8	1.568
		3	287.4	70.4	1.273
	N 1s	1	397.7	53.2	1.055
		2	398.5	36.4	1.541
		3	400.0	10.4	1.228
Co/P-C_3N_4-2%,	C 1s	1	283.8	7.1	0.938
		2	284.4	12.9	1.458
		3	287.6	79.0	1.258
	N 1s	1	397.9	65.7	1.149
		2	398.9	23.6	1.405
		3	400.3	10.6	1.214
	P 2p	1	132.1	42.78	1.377
		2	132.8	37.77	1.185
		3	133.8	19.45	1.298
